# Case of Retained Placenta

**Published:** 1857-08

**Authors:** F. R. Bailey

**Affiliations:** Joliet, Ill.


ARTICLE III.—*Case of Retained Placenta.* By F. R. Bailey, M. D., Joliet, Ill.

I was called February 21st, about 2 A. M., to attend a lady laboring under profuse uterine hemorrhage, attended with severe pain in the back, etc. I learned that she was about three months and a half advanced, and endeavored at once to control the flooding, and relieve the pain, so as to prevent abortion. It was of no avail, however, for in a short time the fœtus was expelled.

The patient being considerably exhausted, it was necessary to administer stimulants and opiates, but as soon as 9 o'clock in the morning she was very comfortable, with no more appearance of flooding than is usual in such cases.

After removing the fœtus, I waited a short time for the expulsion of the placenta; but there being no effort of the uterus in that direction, made a vaginal examination, and could find nothing of it. Being very certain that it could not have escaped my notice, if expelled, and that it must still be "in utero," I watched closely for a day or two, in order to obviate hœmorrhage.

Nothing was seen of it, however, and my patient was in a few days about the house as usual. The conclusion was, that it had escaped without having been seen by any one. After about two weeks from the occurrence of the abortion, I was called in, and found her complaining of a fullness in the pelvic region, and was feeling as though there might still be another fœtus unexpelled. The lochial discharge still continued, but to a moderate degree; and as the bowels were constipated, I gave a dose of black draught, which operated well, and all the sense of fullness, together with any apprehensions before mentioned, subsided. From this time she improved rapidly, and was soon able to attend to her household duties, and to walk about the city. The lochia, as I subsequently learned, continued, but no more profuse than is common in many cases.

I heard nothing more of my patient until the 6th of April, about one o'clock A. M., when I was hastily summoned, with the assurance that she was "flowing to death." In a few moments I was at the bedside, and found her pulseless and gasping. A great quantity of blood had escaped, and it was still flowing like a stream. I immediately gave a dose of opium and acetate of lead, and passed small lumps of ice into the vagina. In a few minutes the flowing stopped, but it was five or six hours before she was able to speak above a whisper. Nothing could be detected by examination by which to conclude that there was anything still unexpelled from the uterus, and as the flow had stopped, I concluded to give sul. quinine in liberal doses, to prevent a recurrence at the same time on the succeeding morning.

About twenty-six hours from its commencement a slight flow occurred; but it was soon stopped, by means of cold water applied to the abdominal region. She complained of extreme soreness in the back, but no pain.

On Thursday, the 9th, about 6 o'clock P. M., after severe pain for a short time, with some flowing, a placenta, in a state of complete putrefaction, was expelled, and to the great satisfaction of all parties. From this time, by the use of tonics and nutritious food, she rapidly improved in health, and is now able to be about.

*Query*—Is it not unusual for a woman to be able to walk about town, and feel tolerably well, with a retained placenta ?

